# A novel approach for simultaneous detection of the most common food-borne pathogens by multiplex qPCR

**DOI:** 10.17305/bb.2022.8693

**Published:** 2023-08-01

**Authors:** Emir Hodzic, Aida Glavinic, Cara Wademan

**Affiliations:** 1 School of Veterinary Medicine, University of California, Davis, CA, USA; 2 Faculty of Veterinary Medicine, University of Sarajevo, Sarajevo, Bosnia and Herzegovina

**Keywords:** Food-borne pathogens, multiplex quantitative polymerase chain reaction (qPCR), live and dead bacteria, sample concentration.

## Abstract

Food contaminated with bacterial pathogens is a great threat to human health and food spoilage, having an impact on public health and the food industry. Research in food safety seeks to develop a practical, rapid, and sensitive detection technique for food-borne pathogens. In the past few decades, real-time quantitative polymerase chain reaction (qPCR) has been developed, and multiplex qPCR is a preferred feature. Multiplex qPCR enables the simultaneous amplification of many targets of interest in one reaction by using more than one pair of primers. In this study, we have developed and evaluated a hydrolysis (TaqMan) probe-based system for simultaneous detection of eight of the most common food-borne pathogens in a single-step procedure by multiplex qPCR. A multicolor combinational probe coding (MCPC) strategy was utilized that allows multiple fluorophores to label different probes in combinatorial manner. This strategy enabled simultaneous detection, identification, and quantification of targeted genes. The efficiency of the individual qPCR reactions for each target gene had values comparable to those established for multiplex qPCR, with detection limits of approximately < 10 copies of DNA per reaction. Pathogen load helps to predict bacteriological quality status in food products and serves to validate the efficiency of procedures to minimize or eliminate their presence, so newly developed multiplex qPCR was quantitative for each pathogen. During sample preparation, a step to concentrate the target organism from a relatively large sample size, remove all potential PCR inhibitors, and yield samples in a volume suitable for qPCR was incorporated.

## Introduction

Food contaminated with bacterial pathogens is a great threat to human health with over nine million food-borne illnesses acquired in the United States alone [1]. Historically, food safety policy analysis in the United States has relied on cost-of-illness estimates that are limited to costs of medical treatment, productivity losses due to absenteeism, and varying approaches to mortality assessments [2]. The enhanced cost-of-illness model replaces the productivity loss estimates with a more inclusive pain, suffering, and functional disability measure based on monetized quality-adjusted life year estimates. The estimated annual cost of food-borne illness in the United States is between $55.5 billion and $93.2 billion [3]. Moreover, food spoilage, as an outcome of microorganism’s outgrowth that results in biochemical activity that changes foods or beverages to become undesirable or unacceptable for consumption, has had a significant economic impact on the food industry [4, 5].

It is estimated that foods consumed in the United States that were contaminated with 31 known etiological agents of food-borne diseases caused between 9.4 and 76 million illnesses, 56,000 hospitalizations, and over 1300 deaths each year [6–8]. More than 250 known diseases are transmitted through food, caused by bacteria, parasites, viruses, toxins, metals, and prions [6]. *Campylobacter, Salmonella*, *Escherichia coli* O157:H7, *Shigella, Vibrio, Listeria,* and *Yersinia* have been recently reported as the major causative agents of food-borne diseases [8–11].

Research in food safety and consumer protection seeks to develop a practical, rapid, and sensitive detection technique for food-borne pathogens. Current methods primarily rely on enrichment and culture procedures for pathogen detection, however, both techniques can take many days to obtain a definitive result. Even though traditional culturing methods are considered the gold standard since they are highly accurate and reliable, their major drawback is that they involve multiple steps, making the process lengthy. Therefore, culturing methods are unattractive from the food industry perspective, which desires more rapid testing. An assay that has the potential to speed the overall analysis culture-based methods with the additional ability to quantify food-borne pathogens is a solution to make food safety surveillance more effective. Even though many methods of detection are available, food and environmental microbiologists usually have to choose between enumeration and identification without the option of both [12]. In addition to culture- and colony-based methods, other methodologies have been utilized for the detection of food-borne pathogens, such as immunology-based methods and biosensors [13]. Recently, new advanced molecular diagnostic techniques for the detection of food-borne pathogens have been developed. Those techniques include polymerase spiral reaction assays [14] and loop-mediated isothermal amplification assay [15], among others.

More rapid detection of these pathogens is of great importance to ensure food safety. An assay that has the potential to speed the overall analysis culture-based methods with the additional ability to quantify food-borne pathogens is a solution to make food safety surveillance more effective. The United States Department of Agriculture, Agriculture and Food Research Initiative Competitive Grants Program identifies one priority area as “innovative studies which seek to quantify the effectiveness of the existing intervention or management strategies in reducing pathogen loads from farm-to-fork.” Development of these assays would provide a valuable and unique tool to assess food contamination and mitigation measures.

Real-time quantitative polymerase chain reaction (qPCR) combined with the use of viability dyes, recently introduced for the purpose of distinction between live and dead pathogens, fulfills all these requirements [16]. In the past several years, molecular techniques have been developed for the detection of food-borne pathogens, especially qPCR [5, 17, 18]. These techniques have revolutionized analysis in the field of microbiology allowing the detection of pathogenic microorganisms in food, without the necessity of classical isolation and identification that is often labor intensive and time consuming [16, 19]. Molecular techniques have reduced time to result (TTR) which represents one of two key performance standards which are often used to evaluate a detection tool along with assay sensitivity, the ability to detect as little as one target cell per 25–325 g sample [20]. However, the trend has been to develop methods for the detection of multiple pathogens so that food testing can be economical. Multiplex qPCR enables simultaneous amplification of many targets of interest in one reaction by using more than one pair of primers. Even though multiplexing is a preferred feature, few efficient multiplex assays have been established [21, 22]. According to Hu et al. [20], up until recently, a method for simultaneous identification of eight food-borne pathogens in a single reaction has been developed using modified molecular beacons. Such approach is capable of simultaneously detecting *L. monocytogenes, S. enterica subsp. enterica, E. coli O157, V. parahaemolyticus, V. vulnificus, C. jejuni, E. sakazakii, and Shigella* spp. [19].

The specific goal of our research is to develop and optimize a more sensitive and rapid system for simultaneous detection of eight of the most common bacterial food-borne pathogens in a single-step procedure by multiplex qPCR. Multiplex qPCR enables simultaneous amplification of many targets of interest in one reaction by using more than one pair of primers. There is a limitation to pursuing multiplexing since current commercial thermocyclers can only differentiate three to four colors of dye. Therefore, up to four PCR reactions need to be optimized for similar amplification efficiencies to produce a reliable multiplexed reaction. Speicher et al. [23] developed multiplex-fluorescence in situ hybridization (FISH) of six fluorophores with different excitation wavelengths that provided a high degree distinction between all possible pairs. Using four fluorophores with different excitation wavelengths to label qPCR probes, we have demonstrated simulation detection of eight food-borne pathogens. Additional challenges for multiplex PCR of food samples are low levels of contaminating pathogens, PCR inhibitors, detecting dead pathogens, splitting sample DNA, and high sample mass compared to amplification volumes. The methodology we used in the study is simple, sensitive, and reproducible and can be used efficiently in the detection of food-borne pathogens.

## Materials and methods

### Food-borne bacterial pathogens

The most common food-borne pathogens reported in the United States, *Bacillus cereus, C. jejuni, E. coli* O157:H7, *L. monocytogenes, Salmonella* spp., *Shigella* spp., *Staphylococcus aureus,* and *Yersinia enterocolitica* [10], were used in the study. All eight reference bacterial pathogens were obtained from a culture collection grown by the Microbiology laboratory at the University of California, Davis. A pure culture of each bacterial pathogen was grown on adequate solid medium as follows: *E. coli* and *Shigella* spp. were cultured overnight at 37 ^∘^C on MacConkey agar, *Salmonella* spp. at 37 ^∘^C overnight on Brilliant-Green agar, *B. cereus* at 37 ^∘^C overnight on Nutrient agar, *L. monocytogenes*, and *S. aureus* at 37 ^∘^C for 24 h on Trypticase soy agar with 5% sheep blood, *Y. enterocolitica* at 30 ^∘^C for 48 h on MacConkey agar, and *Clostridium perfringens* at 35 ^∘^C for 24 h under anaerobic conditions on Blood agar. Growth of each bacterial pathogen was evidenced by the colonial growth on the agar surface, with characteristic surface texture, transparency, and color. Verification of each isolate was performed by use of qPCR analysis.

### Food samples

Ten food samples were used for testing of eight food-borne pathogens. The tested food samples include beef, sheep, pork, chicken, eggs, cheese, milk, fish, vegetables, and fruit. Prior to spiking food samples with food-borne pathogens, 1 g of each solid sample or 1 mL of liquid sample was analyzed using enrichment culture to confirm the absence of natural contamination. Following confirmation, 10 g or 10 mL of solid or liquid food sample, respectively, were contaminated with either 10^1^ colony-forming units (CFUs), 10^2^ CFU, or 10^3^ CFU of cultured food-borne pathogens in an exponential growth phase. As negative controls, food samples were inoculated with sterile saline. Contaminated food samples were subject to rapid separation and concentration, as described below.

### Extraction of DNA

Each bacterial strain was harvested from agar plates, suspended in sterile PBS, centrifuged at 12,000 ×*g*, and pellets were washed in PBS and used for DNA extraction.

It is difficult to detect low presence of food-borne pathogens among large number of background microflora in food samples. To enhance the separation and extraction of bacterial DNA from food samples, a method for separation and concentration of food-borne pathogens, slightly modified from that described previously [24, 25], was utilized. Briefly, samples homogenized in Geno/Grinder 2010 (SPEXSamplePrep, Metuchen, NJ) were subjected to filtration, high-speed centrifugation at 16,000 ×*g* for 5 min, and sedimentation.

Total nucleic acid from bacteria cultures and concentrated food samples was extracted with a QIACube HT system (Qiagen, Valencia, CA, USA) according to the manufacturer’s instruction for tissue and bacteria. This procedure is also intended to clean away inhibitors and concentrate the bacteria in sample volumes suitable for PCR. The concentration and purity of extracted DNA were determined by measuring the *A*_260_ and *A*_280_. The extracted nucleic acid was stored at −80 ^∘^C until use.

**Table 1:** Oligonucleotides primers and hydrolysis internal probes for amplification of food-borne pathogens used in singleplex and multiplex qPCR

**Bacterial strains**	**Target gene**	**Amplicon position**	**Sequence 5’-3’**
*Bacillus cereus*	*ssPE*	45-211	F-CAACTTCTGGTGCTAGCATTCAA
Accession #DQ146919			R-TGCAAATTCAGTACCATAACTAGCG
			p1-**FAM**-AAGCTTCTGGTGCACAAA (MGB)
			p2-**VIC**–AAGCTTCTGGTGCACAAA (MGB)
*Campylobacter jejuni*	*fabZ*	415-578	F-GCAGGACATGTAGAGCTTGGAGA
Accession #AY531523			R-CTTCTAATACTTGCACGATTTCCTTCT
			p-**TET-**CAGGAGCAAGTGCAC (MGB)
*Escherichia coli* O157:H7	*rfbE*	681-845	F-GGTCTCAATTCTAACTAGGACCGC
Accession #JN578671			R-CCACGCCAACCAAGATCC
			p-**VIC**-CACACGATGCCAATGT (MGB)
*Listeria monocytogenes*	*hylA*	104-266	F-CAATTTCATCCATGGCACCAC
Accession #HM589604			R-CTTGGCGGCACATTTGTCA
			p1-**FAM**-ACACGCGGATGAAA (MGB)
			p2-**TET**-ACACGCGGATGAAA (MGB)
*Salmonella* spp.	*invA*	308-484	F-CGCTCTTTCGTCTGGCATTATC
Accession #M90846			R-CTGAACCTTTGGTAATAACGATAAACTG
			p-**FAM**-TTATTGGCGATAGCCTGG (MGB)
*Shigella* spp.	*ompA*	429-596	F-GGAATACCAGTGGACCAACAACA
Accession #AY305875			R-TTCAGAGTGAAGTGCTTGGTCTG
			p-**NED-** TAGTTGCTCCAGCTCC(MGB)
*Staphylococcus aureus*	*scaD*	300-465	F-TGGTTTAGGTGCAAGCTACAGC
Accession #HM565739			R-TTACCACCTACACGATCAAATACGTAG
			p1-**FAM-**CATCATCAAATGGCCGTTC (MGB)
			p2-**NED–**CATCATCAAATGGCCGTTC (MGB)
*Yersinia enterocolitica*	*sat*	522-688	F-AGTGGTCACGCGCGATGT
Accession #AF170730			R-ACCGGCAACGATCAGGTGTA
			p1-**TET**- AATCCCGCAACACTG (MGB)
			p2-**VIC**- AATCCCGCAACACTG (MGB)

qPCR: Quantitative polymerase chain reaction.

### qPCR analysis

For quantitative analysis of DNA extracted from bacteria culture and food samples, qPCR was used and optimized, as previously described [26]. The analytical sensitivity for each target gene DNA was in the range of 1–10^9^ bacterial cells, with a yield of detection close to 95% of the calculated amount of the known target in each sample. All qPCR reactions for each target gene were performed with positive bacterial controls, negative reagent controls, and non-spiked food samples DNA controls. Obtained data were reported as positive or negative, or expressed as the DNA copy numbers per mg of food weight.

For each of the eight food-borne pathogens, two specific primers and one internal, fluorescence-labeled probe were designed with Primer Express software 3 (ThermoFisher Scientific), targeting more conserved genes. Target sequences of each food-borne pathogen were carefully selected to elude homologies to other organisms to assure high specificity of detection. Oligonucleotide PCR primers and hydrolysis internal probes of each target genes used in this study are shown in Table 1. Sequence information for each target gene is available in the GenBank (NCBI). For the development and optimization of sensitive and rapid system for simultaneous detection of eight bacterial food-borne pathogens in a single-well, a multiplex qPCR was established. To monitor the amplification of the target sequences, TaqMan^®^ MGB™ probes that incorporate a 5’ reporter dye (FAM, VIC, TET, NED) and a 3’ nonfluorescent quencher (NFQ) were used. A multicolor combinational probe coding (MCPC) strategy that has been published previously [23] was utilized in the study. This approach utilizes fluorophore combinations in addition to a single fluorophore to label probes, allowing *N* types of fluorophores to label different probes in a combinatorial manner. Thus, up to 15 probes can be labeled using 4 different fluorophores, and 15 targets can be detected on a 4-color qPCR thermocycler. Using MCPC, eight food-borne pathogens can be accurately identified at the species level in a single qPCR. Four probes were each labeled with a single reporter, and another six each were mixed labeled with various combinations of two reporter dyes (mixtures of two probes having the same oligonucleotide sequence, but each labeled with a different reporter). Each individual assay was validated and optimized as a singleplex to achieve an efficiency of 90%–100%. Then multiplex assays were optimized, to yield a similar efficiency, as a singleplex assay. The optimization process examined the equimolar primer mixture to find the optimal individual primer concentration, multiplex cycling conditions, and multiplex reaction components (deoxynucleotide triphosphates, buffer, and polymerase). The specificity of each assay was determined using DNA templates in duplicate from other pathogens.

Each qPCR reaction contained 20× primers and a probe for the respective qPCR assay with a final concentration of 400 nM for each primer and 80 nM for the MGB probe and commercially available PCR mastermix (TaqMan^TM^ Universal PCR Mastermix, ThermoFisher Scientific) containing 10 mM Tris–HCl (pH 8.3), 50 mM KCl, 5 mM MgCl_2_, 2.5 mM deoxynucleotide triphosphates, 0.625 U AmpliTaq Gold DNA polymerase per reaction, 0.25 U AmpErase UNG per reaction, and 5 µL of the diluted DNA sample in a final volume of 12 µL. The samples and positive and negative controls were placed in 384 well plate and PCR amplifications were performed in the ABI Prism 7900HT Fast Real-Time PCR system (Applied Biosystems). Amplification was done under the following thermal cycle conditions: 2 min at 50 ^∘^C to, 10 min at 94.5 ^∘^C, followed by 40 cycles of denaturation at 97 ^∘^C for 30 s, and annealing and extension at 59.7 ^∘^C for 1 min.

The amplification efficiency (*E*) of all assays was calculated from the slope of a standard curve generated from 10-fold serial dilutions run in triplicate, using the formula *E* ═ 10^(−1/slope)^ −1. Estimated PCR efficiency should be reported from replicated calibration curve, and optimal efficiency is achieved at a slope of −3.32 [27]. The coefficient of determination (*R*^2^) was also calculated to assess the validity of the linear regression. To assess the repeatability of qPCR assays, intra- and inter-assay coefficient of variability (CV) were calculated [28]. To obtain intra- and inter-assays CV, samples were run on a same plate or multiple assay plates, respectively. The CV was performed for singleplex qPCR and multiplex qPCR.

For absolute quantification, a standard curve of each individual pathogen was constructed from known CFU concentration of bacterial cells. Standard curves were greatly reproducible allowing the generation of highly specific, sensitive, and reproducible data. The standard curves were constructed from the diluted standard template and then can be used to determine the quantity of targeted gene in the unknown sample by interpolation. Quantification with external standards was carefully optimized for precision (replicates in the same kinetic PCR run—intra-assay variation) and reproducibility (replicates in separate kinetic PCR runs—inter-assay variation) to understand the limitations within the given application.

### Statistical analysis

Statistical analysis of qPCR data was performed using independent samples *t*-test or one-way analysis of variance, followed by multiple pairwise comparisons by Tukey’s honestly significant difference (HSD) test (SPSS 16.0 for Mac; SPSS Inc., Chicago, IL, USA).

## Results

### Specificity and efficiency of qPCR assays

To facilitate specificity, a genomic region of each food-borne pathogen to be amplified was assessed for homologous regions between sequences of other microorganisms by utilizing the Basic Local Alignment Search Tool (BLAST, NCBI). The GenBank BLAST analysis revealed that each assay is highly specific and sequence identity and similarity between them and other microorganisms ranges from 11% to 57%. Amplification specificity is determined by primer length and an annealing temperature. Additionally, for multiplexing, a generated amplicon should be of a similar size. Optimal assay design resulted in similar primer annealing temperatures, as well as in similar amplicon sizes (Table 1). The designed qPCR assays for *B. cereus, C. jejuni, E. coli* O157:H7, *L. monocytogenes, Salmonella* spp., *Shigella* spp., *S. aureus,* and *Y. enterocolitica* amplified homologous, but not heterologous, species-specific amplicons from culture as well as from food samples contaminated with each individual bacterium. The kinetics of qPCR amplification specificity of each assay during thermocycling are directly related to the starting number of DNA copies. The obtained results have shown that all assays were specific to their corresponding target pathogens.

**Figure 1.**
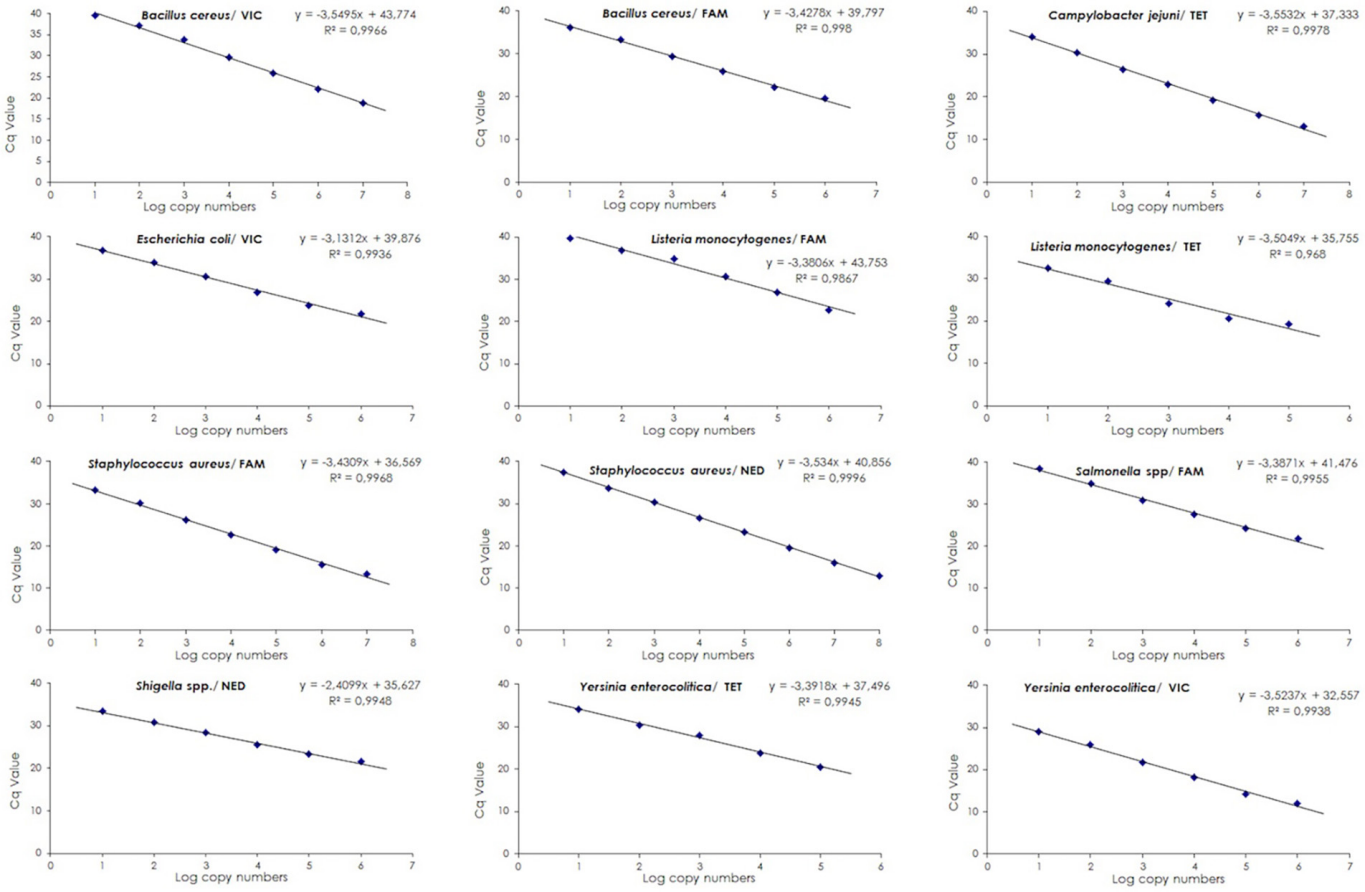
**Validation standard curves analysis of eight qPCR assays sensitivity used in the study**. The *X-*axis displays the log copies/well and the *Y*-axis represents the measured Cq value. qPCR efficiency is determined by using the fold serial dilutions of extracted gDNA. Cycle efficiency is regressed against cycle fluorescence that estimated the *y*-intercept and the slope. The *y*-intercept of the regression line yields the maximal efficiency estimate. qPCR: Real-time quantitative polymerase chain reaction; Cq: Quantitative cycle; R^2^: Coefficient of determination.

**Figure 2.**
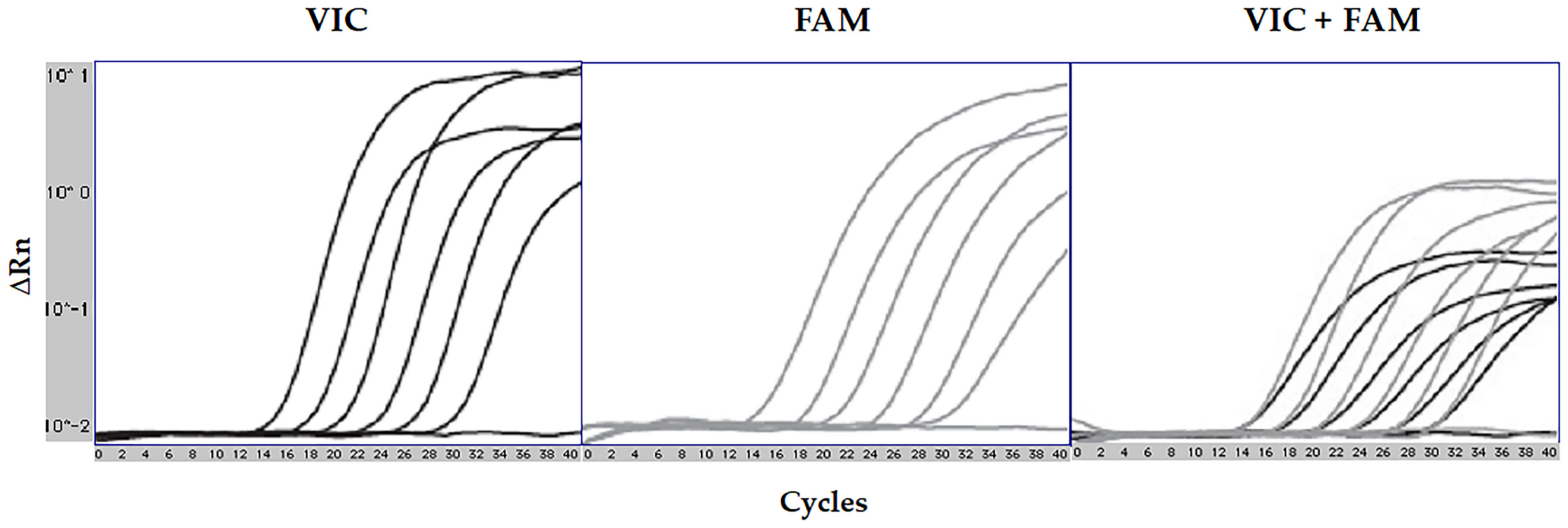
**Three separate qPCRs for *B. cereus* were performed using VIC-labeled probe, FAM-labeled probe, and both VIC and FAM mix-labeled probes.** Linear view of analyzed qPCR results was depicted in three graphs. Purified DNA templates were 10-fold serially diluted from 10 ng to 0.1 pg and water was used as a negative control. Note that among the entire range of template concentrations, no obvious difference in Cq values among three reactions was observed, but slightly lowered fluorescence intensity occurred with the dual-color probe reaction. qPCR: Real-time quantitative polymerase chain reaction; Cq: Quantitative cycle.

**Figure 3.**
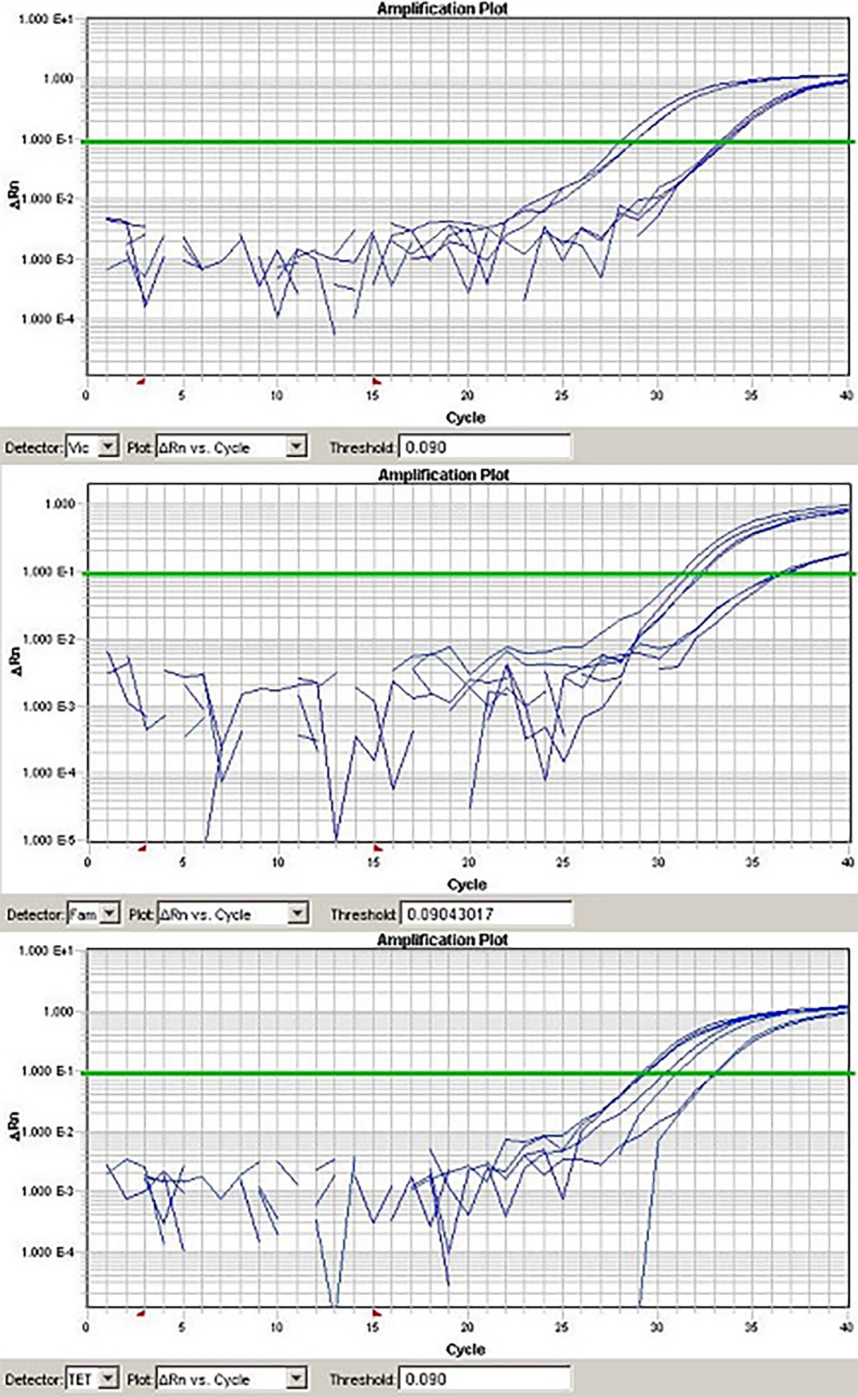
**Logarithmic view analysis of multiplex qPCR containing *B. cereus* (VIC, TET) and *C. jejuni* (TET) specific assays.** DNA was purified from food samples contaminated with both *B. cereus* and *C. campylobacter* cultured organisms at different CFU. Each graph represents a separate PCR analysis for the corresponding fluorophore. CFU: Colony forming unit; qPCR: Real-time quantitative polymerase chain reaction.

The efficiency of qPCR assays is critical for the accuracy of data obtained from PCR. Standard curves for each target gene were generated from 10-fold serial dilutions by linear regression analysis, plotting the quantitative cycle (Cq) values in the *Y*-axis versus the logarithm of the starting DNA dilutions in the *X*-axis (Figure 1). All assays used in the study were determined to have an efficiency between 91% and 104% (Table 2). The assessment of qPCR efficiency for *B. cereus* is depicted in Figure 2; no obvious difference in Cq values among three different reactions was observed. Similar findings were determined for all other assays. Based on the amplification efficiencies, detection limits were approximately < 10 copies of DNA per reaction. Assessment of the efficiency in multiplex qPCR utilizing all eight assays resulted in similar amplification efficiencies for their respective target genes. Multiplex qPCR has shown to be independent of the input amount of DNA copies (Figure 3).

**Table 2:** PCR efficiency of each qPCR assay used for multiplexing

**Bacterial strains**	**Efficiency**	**Fluorescent reporter**
*Bacillus cereus*	91.7%	VIC
	95.8%	FAM
*Campylobacter jejuni*	91.2%	TET
*Escherichia coli* O157:H6	103.6%	VIC
*Listeria monocytogenes*	97.6%	FAM
	96.2%	TET
*Staphylococcus aureus*	91.9%	NED
	95.6%	FAM
*Salmonella* spp.	97.4%	FAM
*Shigella* spp.	95.0%	NED
*Yersinia enterocolitica*	96.1%	VIC
	97.2%	TET

qPCR: Real-time quantitative polymerase chain reaction.

### Intra- and inter-assay variation

To validate the accuracy and reproducibility of qPCR intra- and inter-assay variation, reactions were run in three repeats. Fluorescent signals were collected during the annealing temperature and Cq was calculated and exported with a threshold of 0.09 and a baseline of 3–12 for the genes of interest. For singleplex qPCR, the intra-assay CV determined for 10 replicates was in the range of 0.42%–2.8%, and for multiplex qPCR was in the range of 1.2%–4.5%. The inter-assay CV was determined to be 1.8%–4.9% and 3.7%–7.2% for singleplex and multiplex qPCR, respectively. It is generally acceptable that intra-assay CV should be below 10%, and for inter-assay, the CV should be below 15%.

### Sensitivity of a singleplex qPCR assay for each food-borne pathogen

Genomic DNA of each pathogen was serially diluted by limiting dilution to determine the absolute sensitivity and reproducibility of qPCR with each assay. The results were consistently positive at the lowest input amount of genomic DNA with the corresponding assay for all 8 species, revealing the analytical sensitivity range from 10^1^ to 10^9^ copy numbers of targeted genes. The results also revealed that a yield of detection was around 95% for *B. cereus, E. coli* O157:H7, *L. monocytogenes, Salmonella* spp., *Shigella* spp., and *S. aureus*, and around 92% for *C. jejuni* and *Y. enterocolitica*.

### Quantitative detection of food-borne pathogens

The copy number of each food-borne pathogen target gene was expressed per weight of assessed food samples. The dynamic range of each pathogen’s standard curve was up to 9 orders of magnitude from < 10^1^ to > 10^10^ bacterial cells. The target genes of each pathogen were amplified in contaminated food samples, and the copy numbers were 84.3% to 91.5% of the input. The expected yield of extracted DNA from food samples was around 80%. The loss of 8.5%–15.7% of spiked bacteria was within the expected range.

### Detecting low presence of food-borne pathogens

Food samples tend to be less homogeneous and contain lower levels of target organisms, so it is important to concentrate the target organism from a relatively large sample size. Also, various food samples make PCR particularly vulnerable due to the presence of inhibitors. To test the potential of sample preparation by utilizing the separation and concentration approach, minced meat (beef, sheep, pork, and chicken), eggs, cheese, milk, fish, vegetables, and fruit were contaminated in parallel with different spiked levels of *B. cereus, C. jejuni, E. coli* O157:H7, *L. monocytogenes, Salmonella* spp., *Shigella* spp., *S. aureus,* and *Y. enterocolitica*. The obtained results revealed that the detection limit for *E. coli* O157:H7 in all contaminated food samples was 10^1^ CFU (high sensitivity) per 10 g/10 mL of sample. High detection sensitivity was also detected for *S. aureus* in eight contaminated samples, *Salmonella* spp. in seven samples, and *Shigella* spp. in six samples. Medium detection sensitivity of 10^2^ CFU per 10 g/10 mL of food sample was detected for other investigated pathogens in all (*C. jejuni*, *Y. enterocolitica*) or most of the assessed samples (*B. cereus*, *L. monocytogenes*) (Table 3).

### Multiplex qPCR

Developed and optimized multiplex qPCR was assessed for the detection of eight food-borne pathogens in contaminated food samples in a single-step procedure. Using MCPC, eight food-borne pathogens can be accurately identified at the species level in a single qPCR reaction (Table 4). Multiplex qPCR successfully detected *B. cereus, E. coli* O157:H7, *Salmonella* spp., *Shigella* spp., and *S. aureus* in all examined food samples. *C. jejuni, L. monocytogenes,* and *Y. enterocolitica* were not detected in 1–2 examined samples.

**Table 3:** Detection sensitivity of spiked food-borne pathogens in different food samples after separation and concentration process

	**Food samples**									
**Bacterial strains**	**Beef**	**Sheep**	**Pork**	**Chicken**	**Eggs**	**Cheese**	**Milk**	**Fish**	**Veg**	**Fruit**
*B. cereus*	+++*	++	++	+++	++	++	++	+++	++	++
*C. jejuni*	++	++	++	++	++	++	++	++	++	++
*E. coli* O157:H6	+++	+++	+++	+++	+++	+++	+++	+++	+++	+++
*L. monocytogenes*	+++	++	++	++	++	++	++	++	++	++
*S. aureus*	+++	+++	+++	+++	+++	+++	+++	+++	++	++
*Salmonella* spp.	+++	+++	+++	++	++	+++	+++	+++	+++	++
*Shigella* spp.	+++	+++	+++	+++	+++	++	++	+++	++	++
*Y. enterocolitica*	++	++	++	++	++	++	++	++	++	++

*Different levels of spiked pathogens: +++ 10^1^ CFU (high sensitivity), ++ 10^2^ CFU (medium sensitivity), + 10^3^ CFU (low sensitivity). CFU: Colony forming unit; Veg: Vegetable.

**Table 4:** Detection of food-borne pathogen in food samples contaminated with different microorganism by singleplex and multiplex qPCR

	**Tested food**	**Multiplex**	**Singleplex**
**Pathogen**	**samples**	**detected**	**detected**
*Bacillus cereus*	10	10	10
*Campylobacter jejuni*	10	8	10
*Escherichia coli* O157:H8	10	10	10
*Listeria monocytogenes*	10	8	10
*Staphylococcus aureus*	10	10	10
*Salmonella* spp.	10	10	10
*Shigella* spp.	10	10	10
*Yersinia enterocolitica*	10	9	10

## Discussion

The increase of public interest in food safety has led to intensified development of new and rapid methods for the detection of food-borne pathogens [16]. The main objectives of these methods are to verify nonexistence of food-borne pathogens in food products and to confirm their safety, as well as to assess the eventual pathogen’s load as an indicator of proper hygiene and determine the quality of food products [13]. In the past several years, molecular techniques have been developed for the detection of food-borne pathogens. qPCR is one that has been shown to be an especially reliable, rapid, robust, and economical technique. Compared to other available detection methods, it has one of the shortest TTR. This is a preferable feature of any method used for microbiological testing of food-borne pathogens, especially during outbreaks when it is of crucial importance to obtain results in a timely manner [11, 29]. Upon identification, a proper and prompt reaction to the outbreak can reduce morbidity and mortality.

In this study, we have developed and evaluated hydrolysis probe-based qPCR assays for simultaneous detection of eight of the most common food-borne pathogens in the United States. As a target for each pathogen, more conserved genes, mostly chromosomally located, were selected. It has been shown that chromosomally located genes are more stable in comparison to plasmid ones, which are subject to loss from the bacterial cells [30]. The stability of selected target genes of each food-borne pathogen utilized in the study (*ssPE, fabZ, rfbE, hylA, invA, ompA, scaD*, *sat*) has previously been reported [31–35]. Other studies that utilized hydrolyses probes targeted different genes for *C. jejuni (mapA)*, *L. monocytogenes (prfA)*, *Salmonella* spp. (*hilA*), *Shigella* spp. (*ipaH9.8*), *S. aureus (ebpS)*, *Y. enterocolitica (foxA, ail)* [30, 36]. Selection of different target genes for the detection of pathogens still allows comparison between studies as the genes chosen are conservative fragments of DNA sequences.

This paper describes the development of a multiplex qPCR assay for the detection of eight food-borne pathogens using the MCPC strategy and comparing its performance to singleplex qPCR assays for each pathogen. The MCPC strategy applies fluorophore combinations in addition to a single fluorophore to label probes. This approach resembles the so-called chromosome painting technology [37], and the combination concept allows multiple fluorophores to label different probes in combinatorial manner [23, 38]. We have shown that combinatorial probe labeling for qPCR enables simultaneous detection, identification, and quantification of eight different food-borne pathogens. The developed multiplex qPCR assay is considerably very sensitive, highly specific, robust, and reliable to detect minimum amount of each pathogen in spiked food samples. Current commercial technologies have limited detector capabilities allowing reliable multiplex qPCR for 3–4 differently-labeled probes. Parichehr et al. [35] reported four fluorophore-labeled probes used for simultaneous detection of food-borne pathogens in milk. Barletta et al. [30] used SYBR green multiplex qPCR for detection of *Campylobacter*, *Salmonella*, and *Shigella*; however, this approach had shown to have a low sensitivity for detecting pathogens in spiked samples. Multiplex PCR has been reported in several other studies using different techniques with mixed results [21, 36, 39, 40].

The first step in assembling a multiplex assay is to optimize the individual singleplex qPCR reactions by determining the efficiency of each individual reaction. This was achieved by constructing a standard curve using a series of template dilutions. The efficiency as well as the intra- and inter-assay validation of the individual qPCR reactions for each target gene had values comparable to those established for multiplex qPCR. Based on the amplification efficiencies, detection limits were approximately < 10 copies of DNA per reaction for each target gene. Using a single set of reaction conditions, Liu et al. [36] reported of higher detection limit for some of qPCR assays, suggesting less sensitivity. Similar results were reported by Barletta et al. [30] for the detection limit of *Salmonella, Shigella*, and *Campylobacter*, while using intercalating dye SYBR green, which has not recommended for multiplexing. Huang et al. [38] used multiplex qPCR for the detection of *Salmonella* species, *L. monocytogenes*, and *E. coli* O157:H7 in ground meat and reported the detection limit for each assay to be 2 × 10^2^ CFU/mL. The approach that was used in this study has shown some advantages in comparison to similar studies utilizing multiplex PCR for the detection of pathogenic microorganisms. A potential disadvantage in developing and utilizing multiplex qPCR for the detection of microorganisms is the optimization of primers concentrations in relationship to optimal template DNA concentrations [41]. We have shown that the multiplex qPCR approach resulted in an even amplification of all eight target genes when all DNA templates were mixed and run in the same format as singleplex, which did not compromise the detection sensitivity and specificity.

The results from this study showed that the chosen primers and probes provided specific detection of many specific pathogenic bacteria in both culture medium and artificially inoculated samples of beef, sheep, pork, chicken, eggs, cheese, milk, fish, fruits, and vegetables, suggesting that this newly developed assay is specific and reliable. Therefore, this developed protocol could have important applications for rapid and simultaneous detection and identification of up to eight food-borne pathogenic bacteria in a diverse range of food types.

Despite many advantages, qPCR still faces some drawbacks such as food matrix inhibitors and the inability of the methods to discriminate between viable and dead cells which often results in an overestimation of the target microorganisms [16]. Several studies have introduced methods to distinguish between viable and dead bacterial cells, such as using intercalating dyes (propidium monoazide and ethidium monoazide), implementation of viability PCR, and pre-rRNA analysis [16, 42, 43]. A step to concentrate the target organism from a relatively large sample size, remove all potential PCR inhibitors, and yield samples in a volume suitable for qPCR was utilized. This concentration approach proved to be highly sensitive and sufficiently amplified detectable amounts of each target gene. When lower levels of spiked pathogens (down to 10^1^ CFU per reaction) were tested, all targets were detectable, but Cq values were slightly weaker in several food sample types. This was independent of whether the pathogen was spiked as a single target or in a mix containing all pathogens.

## Conclusion

The goal of this study was to develop and optimize a more sensitive and rapid system for the simultaneous detection of eight of the most common bacterial food-borne pathogens in a single-step procedure by multiplex qPCR. The developed multiplex qPCR was quantitative for each pathogen which intends to predict bacteriological quality status in food products and serve to validate the efficiency of food processing procedures to minimize or eliminate their presence. The use of this multiplex qPCR in routine detection of common food-borne pathogens will reduce time and labor. One of the main advantages of the multiplex qPCR is that it offers the possibility of having only one test to detect eight pathogens, while maintaining a high degree of specificity and sensitivity.
